# Clinical-Epidemiological Characteristics and Outcomes of Latent Tuberculosis Treatment at a Tertiary Center in Central-West Brazil from 2017 to 2019

**DOI:** 10.3390/tropicalmed7120432

**Published:** 2022-12-12

**Authors:** Moara Alves Santa Bárbara Borges, Iago Dib Cunha, Luís Henrique Candini, Vitor Alves de Souza, Paulo Sérgio Sucasas da Costa

**Affiliations:** 1 Institute of Tropical Pathology and Public Health, Federal University of Goiás, Goiania 74605-050, Brazil; 2 Clinical Hospital, Federal University of Goiás, Empresa Brasileira de Serviços Hospitalares, Goiania 74605-050, Brazil; 3 Medicine School, Federal University of Goiás, Goiania 74605-050, Brazil

**Keywords:** anti-tuberculosis agent, epidemiology, HIV, latent tuberculosis infections, therapies

## Abstract

Detailed information concerning latent tuberculosis infection (LTBI) and treatment outcomes is scarce in Brazil. This retrospective cross-sectional study aimed to describe LTB treatment (LTBT) at a tertiary center in Central-West Brazil from 2017 to 2019. We recommended the use of LTBTs before the implementation of a rifapentine-isoniazid (3HP) regimen in Brazil. We conducted a descriptive analysis using chi-square or *t*-tests to assess differences in the proportions and means. Of 79 notified adult patients (males, 68%; median age, 40 (interquartile range, 30–51) years), most people were living with human immunodeficiency virus (PLHIV) (82%) or receiving immunosuppressant medication (15%), and 92% were receiving their first treatment. Isoniazid (INH) for 6–9 months had previously been proposed for 95% of the patients, with only 35% completeness. Four patients treated with rifampicin (4RMP) completed the regimen (*p* = 0.009). Adverse events occurred in 19% of the patients. In this Brazilian tertiary center, the target population for LTBT were young PLHIV patients under immunosuppression with low education levels. However, the INH monotherapy dropout rate was 65%. Therefore, shorter courses, such as 3HP and 4RMP, are promising alternatives. Behavioral aspects, education level, and regimen length can influence the course completion, and further studies are required to evaluate the 3HP regime in Brazil.

## 1. Introduction

Tuberculosis (TB), a chronic infectious disease caused by *Mycobacterium tuberculosis* (MTB), may present in a latent or active form, such as pulmonary or extrapulmonary TB, respectively [1]. Latent tuberculosis infection (LTBI) is defined as a state of persistent response of the organism to an infectious acid-fast bacterium without evidence of clinical manifestation [2].

It is estimated that one-third of the world’s population is infected with MTB, of whom between 5% and 10% will develop active TB during their lifetimes. This percentage is higher in patients with some degree of immunological response impairment, especially those infected with human immunodeficiency virus (HIV), those with chronic diseases, and those undergoing immunosuppressive treatments [3].

The tuberculin test (TT) and the interferon gamma release assay (IGRA) test are most commonly used to diagnose LTBI; however, they are not accurate. TT is typically the method of choice for screening LTBI owing to its greater accessibility and low cost. Despite its high cost, IGRA has shown a better performance in immunodeficient individuals and greater specificity [4].

The 2018 World Health Organization (WHO) guidelines recommend screening for LTBI and prescribing preventive treatment for populations at an increased risk of developing active TB. These guidelines advocate isoniazid (INH) administration for 6 months (6INH) and 9 months (9INH) for adults and children living in countries with a high or low incidence of TB. As a recent alternative, regimens with daily doses of rifampicin (RMP) for 4 months (4RMP), weekly doses of INH and rifapentine for 3 months (3HP), and daily doses of INH and RMP for 3 months (3RI) can also be used [5].

By 2021, in Brazil, the available LTBI treatments (LTBT) were 6INH, 9INH, and 4RMP under specific conditions. Several factors may influence therapeutic choices, such as patient comorbidities, laboratory test results, possible adverse events, and the expected adherence to treatment. In July 2021, the Brazilian public health system changed its preferential regimen for LTBT, incorporating 3HP, which has several advantages, such as a decreased treatment time, an increased interval between doses, the convenience of directly observed treatment, and a reduction in the storage and logistics costs [6,7].

Despite its relevance to healthcare, there is a substantial lack of information on this topic, and gaps in the cascade of care must be mitigated. Therefore, we aimed to characterize the epidemiological profile, therapeutic choices, and outcomes of patients treated for LTBI in a tertiary center in Central-West Brazil prior to the incorporation of the shortened treatment duration with 3HP.

## 2. Materials and Methods

### 2.1. Study Design

This was a retrospective cross-sectional study.

### 2.2. Population and Location

Patients with an indication for LTBT who attended a tertiary university center located in Goiânia, the capital of Goiás State in Central-West Brazil, which is a regional referral center for the specialized care of HIV/acquired immunodeficiency syndrome (AIDS), onco-hematology, and rheumatology, during 2017, 2018, and 2019 were eligible for inclusion in this study.

### 2.3. Inclusion Criteria

We included patients aged ≥18 years who met at least one of the following WHO and Brazilian Ministry of Health criteria for LTBT [5,6]:

(1) People living with HIV/AIDS (PLHIV) with:
  - CD4^+^ T lymphocyte count (CD4^+^ TL) of <350 cells/mm^3^, regardless of TT;
  - A TT of ≥5 mm; IGRA-positive;
  - A plain chest radiograph showing a TB scar without prior treatment;
  - A history of pulmonary TB contact.
(2) Patients with a TT of ≥5 mm or who were IGRA-positive with the following risk factors:
  - Treatment with tumor necrosis factor-alpha (TNF-α) inhibitors or other immunosuppressive therapies, including corticosteroids (prednisone > 15 mg/day for >1 month);
  - A history of pulmonary TB contact;
  - On pre- or post-transplantation immunosuppressive therapy.
(3) Patients with a TT of ≥10 mm or who were IGRA-positive with any of the risk factors below:
  - Silicosis, head and neck or hematological neoplasms, renal failure on dialysis, diabetes mellitus (DM), low weight, active smoking, and isolated calcification identified on plain chest radiography.
(4) Patients with TT conversion (10 mm increment from the first to the second measurement).

### 2.4. Exclusion Criteria

We excluded patients who had confirmed active TB or had a plain chest radiograph highly suggestive of TB that was not ruled out at the time of notification, as well as patients who did not have sufficient medical record information for an adequate analysis of the selected variables.

### 2.5. Data Collection

Information was obtained from notification forms provided by the epidemiological surveillance service, and complementary data were accessed through medical chart reviews. Drug dispensing information was provided by a pharmacy service.

The variables collected were as follows:

- Sociodemographic data, including sex, age, self-declared ethnicity, and educational level;
- The patients’ daily habits and health history, such as smoking, previous Bacillus Calmette–Guérin (BCG) vaccination, the presence of comorbidities such as HIV infection, chronic kidney disease, DM, neoplasm, and hematological disease, the transplant schedule, and the use of immunosuppressants (TNF-α inhibitors and corticosteroids);
- Plain chest radiographic description;
- Laboratory test results;
- The proposed treatment regime.

The primary and secondary outcomes were the treatment completeness, evaluated through drug dispense or medical record information, and adverse effects reported in the medical records, respectively.

### 2.6. Statistical Analysis

The data were entered into an electronic Research Electronic Data Capture (REDCap) form and analyzed using the IBM Statistical Packages for the Social Sciences (IBM SPSS, Version 21.0. Armonk, NY, USA: IBM Corp).

We described the measurements of the central tendency (mean and median) and dispersion (interquartile range (IQR) 25% and 75%) for the continuous variables, using a *t*-test to evaluate the between-group differences. In addition, we reported the percentage distribution of the categorical variables by applying the chi-square or Fisher’s exact test to evaluate differences between the proportions. The intention-to-treat analysis considered abandonments, losses to follow-up, deaths, patients who developed active TB during LTBT, and those who did not receive at least 180 doses of INH as the non-completeness of the treatment.

The magnitude of the association between the independent variables and clinical outcomes was estimated at a significance level of *p* < 5% (*p* < 0.05). The crude odds ratio, with the respective 95% confidence intervals (95% CIs), was described in cases of significant association.

### 2.7. Ethical Aspects

This study was conducted in accordance with the guidelines of the Declaration of Helsinki and approved by the Institutional Ethics Committee (protocol code CAAE 48258121.6.0000.5078, on 16 July 2021). Patient consent was waived due to the retrospective nature of the study and the secondary data.

## 3. Results

We evaluated 79 adults who underwent LTBT from 2017 to 2019. The epidemiological characteristics, risk factors, laboratory test results, treatment indications, therapeutic regimens, and outcomes, including the comparison of the variables between the groups who received complete and incomplete proposed treatment, are described in Table 1.

### 3.1. Socio-Epidemiological Aspects

The total sample of 79 patients predominantly consisted of males (n = 54, 68.4%). The median age was 40 years (minimum, 18 years; maximum, 61 years; IQR, 30–51 years). Most of the patients were aged between 30 and 60 years (n = 56, 71%), followed by those aged 18–30 years (n = 21, 26.5%) and those aged >60 years (n = 2, 2.5%). Regarding the years of education, the cumulative percentage of those who did not start higher education (≤12 years of schooling) was 79.1% (34/43), despite the fact that the relevant data were missing for 54.4% of the patients. The most frequent self-declared race was mixed or black (52.7%, 29/55), followed by white (47.3%, 26/55), with 30% (24/79) of the patients having no ethnicity data recorded. The study predominantly recorded new patients (n = 73, 92.4%), with only one re-entry occurring after a change in the treatment scheme (n = 1, 1.3%) owing to hepatotoxicity. Most of the study population (n = 49, 62%) had received a BCG vaccination, and no TB contact was evident in approximately 94% (61/65) of the valid data.

### 3.2. Diagnostic Tests

The diagnostic test information was unspecified in most of the notification forms and patient medical records (n = 44 of 79 patients, 55.7%). TT results were available for 42 out of 44 (95.4%) patients. The median TT was 7 mm (minimum and maximum values of 00 and 28 mm, respectively). Only one case was reported as tuberculin conversion, and only six patients had undergone an IGRA, with no positive results obtained.

Plain chest radiography or computed tomography was performed on 57 (72.1%) patients, of whom 45 (78.9%) had normal results, 3 (5.2%) had TB scars (sequelae), 2 (3.5%) had suspected TB, and 8 (14.0%) had other diseases. The exclusion of active TB was confirmed in 24 (30.4%) of the 79 patients using sputum acid-fast stain or rapid molecular TB tests (GeneXpert MTB/RIF assay; Cepheid, Sunnyvale, CA), which were the methodologies of choice for 20 (83.3%) out of 24 patients, along with isolated mycobacteria culture in the case of 1 (4.2%) patient, and combined methodologies in the case of 3 (12.5%) patients.

### 3.3. Risk Factors for Active TB

The main risk factors for active TB in the 79 study patients were HIV/AIDS (n = 65, 82.3%), the use of immunosuppressants (n = 12, 15.2%), active smoking (n = 12, 15.2%), DM (n = 4, 5.1%), chronic kidney disease (n = 3, 3.8%), and lymphoma (n = 1, 1.3%).

Among the PLHIV cases, the median CD4^+^ TL count was 257 cells/mm^3^ (range, 5–1043 cells/mm^3^). Considering the CD4^+^ LT count, 7 patients (10.8%) had 0–50 cells/mm^3^, 19 (29.2%) had 51–200 cells/mm^3^, 29 (44.6%) had 201–350 cells/mm^3^, and 10 (15.4%) had >350 cells/mm^3^. Among 26 patients with severe immunosuppression (CD4^+^ TL < 200 cells/mm^3^), 18 (70%) did not complete treatment (*p* = 0.700), and 100% of these had <50 cells/mm^3^. The most common antiretroviral therapy (ART) received by the patients with LTBT-HIV was a combined regimen of tenofovir (TDF) 300 mg, lamivudine (3TC) 300 mg, and dolutegravir (DTG) 50 mg (71%, 44/62). Three patients were not on ART. Undetectable viral loads or <50 copies were observed in 49.2% of the patients (32/65), while we observed between 50 and 1000 copies in 13.9% (9/65) and >1000 copies in 36.9% (24/65).

### 3.4. Treatment Indications

**Figure 1:**
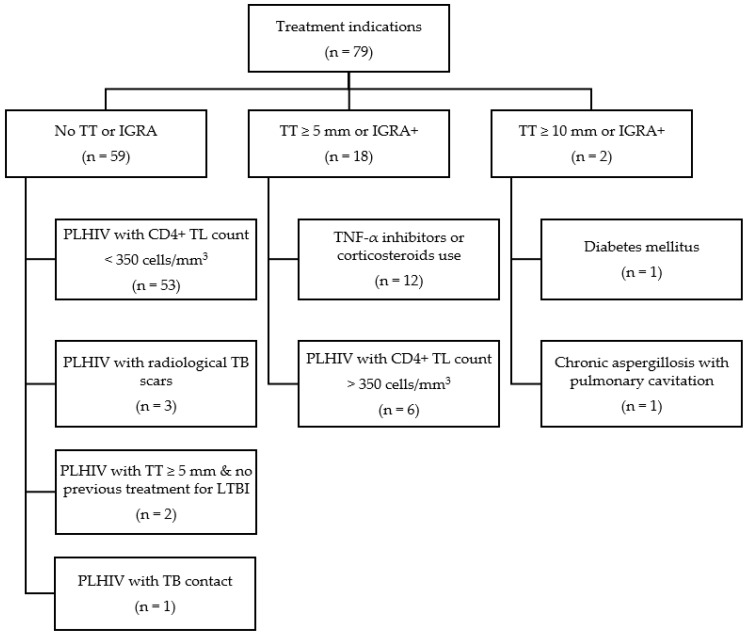
Flowchart of indications for LTBT in a Brazilian tertiary center from 2017 to 2019. CD4^+^ TL, CD4^+^ T lymphocyte; DM, diabetes mellitus; IGRA, interferon gamma release assay; LTBI, latent tuberculosis infection; PLHIV, people living with HIV; TB, tuberculosis; TT, tuberculin test.

The TT or IGRA results were not used as treatment indications for LTBT in 59 of the 79 (74.7%) patients. Of these, 53 (89.8%) were PLHIV and had a CD4^+^ TL count of <350 cells/mm^3^. A TT result of ≥5 mm was found in 22.8% (18/79), and the use of TNF-α inhibitors or corticosteroids was found in 66.7% (12/18) of this latter group, with the remaining 33.3% (6/18) being PLHIV with a CD4^+^ TL count of >350 cells/mm^3^. A TT of >10 mm was found in 2.5% (2/79) of the total sample, with one patient with DM using methotrexate and one patient with a pulmonary cavitation and chronic aspergillosis. Figure 1 presents the treatment indications.

### 3.5. Proposed Treatments

Regarding the proposed treatment, INH 300 mg/day was administered for 6 months (17.7%, 14/79) or 9 months (n = 61/79, 77.2%) and rifampicin 600 mg/day was administered for 4 months (n = 4/79, 5.1%) to four patients who tested HIV-negative. All four patients treated with rifampicin completed the treatment (*p* = 0.009). Overall, 49 (62.0%) of the 79 patients did not meet the minimum target regimen of 180 doses of INH or rifampicin for 4 months.

Two (2.5%) patients died during the proposed treatment, with neither death being related to adverse events following LTBT. Both deaths involved PLHIV with severe immunosuppression (CD4^+^ TL < 200 cell/mm^3^) and opportunistic infection.

**Figure 2:**
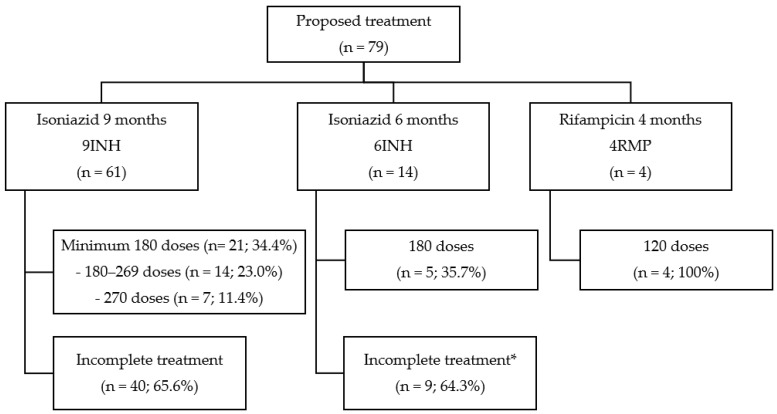
Flowchart of the proposed LTBT and completeness rates in the study center from 2017 to 2019.

Three (3.8%) patients developed active TB after starting LTBT with INH and later switched to TB treatment. One male PLHIV with a CD4^+^ TL of 70 cells/mm^3^ had tuberculous meningitis, diagnosed after four months of LTBT, which was a probable immune reconstitution inflammatory syndrome of a previous occult TB infection. A female with chronic granulomatous mastitis taking corticosteroids and with a TT of 20 mm was initiated on empiric TB treatment following clinical revision. A male patient using a TNF-α inhibitor had fever onset during the seventh month of LTBT, and a renal TB infection was confirmed. Excluding these five patients, the rate of patients who met the minimum treatment goal was 40.5% (30/74). Only two patients underwent supervised treatment, and the treatment supervision information was unknown for 73 (94.8%) of the 79 patients. Figure 2 shows the proposed treatments and their completion rates and includes incomplete treatments in the case of those who received <180 doses of INH, switched to TB treatment, or died.

### 3.6. Adverse Events

Adverse events during LTBT were reported in 15 (19%) of the 79 patients; however, in the case of 46 (57.5%) patients, this information was incomplete or unreported in the medical records. Among these adverse events with possible concomitance, epigastralgia or abdominal pain (n = 8), nausea or vomiting (n = 3), and reddish/orange urine (n = 3) were reported, as well as hepatotoxicity, diarrhea, and peripheral neuropathy in one patient each.

### 3.7. Active TB after Treatment

In 2022, the data obtained from all the medical charts and laboratory records were reviewed and did not indicate any new TB cases.

## 4. Discussion

This retrospective cross-sectional study was undertaken in a referral center for immunosuppressed patients in Brazil, a country with a high risk of tuberculosis infection. The WHO and Brazilian guidelines are periodically updated with the aim of improving the treatment cascade [5,6]. This study aimed to characterize the epidemiological profiles of patients notified of an LTBI diagnosis in a tertiary hospital in Central-West Brazil from 2017 to 2019 and assess the completeness of the proposed treatments according to the existing protocols at the time. However, there was a significant amount of missing data in relation to certain sociodemographic variables and the patients’ daily habits, which may have biased our results.

Men aged 30–60 years with HIV infection accounted for most of the patients reported as having LTBT, followed by those on immunosuppressant mediation. Of the reported patients, <40% had completed the recommended treatment regimen. Among the diagnostic tests, TT was more commonly used, followed by IGRA. Although the 6INH and 9INH were the preferred regimes in the evaluated period, the incompleteness rates were high.

A similar epidemiological study conducted in Southeast Brazil comprising 690 PLHIV who attended a referral center in 2017 showed an association between the occurrence of LTBI and sex. It reported that men with HIV/AIDS were 80% more likely to present with LTBI than women [8]. Another study conducted in Taiwan between 2011 and 2012, comprising 1018 individuals at a high risk of LTBI, showed a significantly higher proportion of males with LTBI (32.6%) than females (25.2%) [9]. These data may be related to sex inequalities in social aspects and behavior, in addition to the reported finding that men are more commonly associated with higher exposure to risk factors such as smoking and HIV/AIDS, with poorer health prevention strategies [8,9].

Our study sample mostly comprised young people, which accords with the patient profile in a multicenter study conducted in Italy, in which the individuals had recently been diagnosed with HIV/AIDS and were IGRA-positive. In that study, the median age of the population diagnosed with LTBI was 37.5 (30–49) years, which was like that recorded in this study [10]. Our study population excluded children and adolescents, and the study site was a tertiary center that offers specialized services for HIV/AIDS, as well as rheumatological and onco-hematological diseases. Therefore, although the findings suggest a higher prevalence of LTBI in middle-aged adults, these data should be interpreted with caution owing to the potential sample selection bias.

Regarding the patients’ education levels, the incomplete data on their schooling meant that it was difficult to determine an accurate education profile. One descriptive study conducted in Southeast Brazil evaluated the epidemiological profiles of LTBI cases seen at a referral center in 2009 and 2010 and reported that 74.5% of the patients had ≤12 years of schooling, indicating a higher prevalence of LTBI in those with a lower level of education [11]. Those with fewer years of schooling, particularly those who had not started higher education, were reported to have generally less favorable socioeconomic conditions, such as living or working in unhealthy locations, rendering them vulnerable to situations that expose them to a higher risk of TB infection [12].

Self-declared race reporting in Brazil remains challenging in terms of evaluation because of the use of non-objective definitions and miscegenation. Moreover, owing to the possibility of sample bias because of the significant amount of missing data, this variable should be interpreted cautiously [13,14].

Our sample was representative of the main recommended criteria for LTBT, such as individuals with a higher risk of progression to active disease, because they either had come into contact with TB cases or lived with an immunosuppressant condition [6,15]. PLHIV had the highest indication for LTBT. This may have been related to the fact that the study was conducted in a reference care center dedicated to this condition, which contributed to the sample selection. HIV immunodeficiency impairs the elimination mechanism mediated through natural killer memory cells and hinders the production of cytokines essential for MTB control [16].

PLHIV are 20 times more likely to develop active TB than those without the infection [2]. In combination, ART and LTBT reduce the risk of disease reactivation by 76% [17]. In Brazil in 2021, only 46.5% of PLHIV with active TB underwent ART during the treatment of this co-infection (TB-HIV), which may suggest difficulty in adhering to the recommended treatments in this population, such as prophylaxis and opportunistic infection therapies [13]. A prospective cohort evaluated 1872 patients who started and completed the stages of the diagnosis and management of LTBI between 2015 and 2019. In this cohort, living with HIV, illiteracy, low socioeconomic status, and black/pardo (brown) race were independently associated with losses in the LTBT cascade of care, especially in the early stages of screening and examination [18].

The LTBT cascade of care for PLHIV remains a challenge. Frequent dropouts at all the stages of care, from the selection of individuals who require LTBI testing to the notification of positive cases, medical evaluation and prescription, and the completion of treatment, represent a shortcoming in the management of this infection worldwide and in Brazil, especially in the public health system. In addition, individuals on HIV immunosuppression treatment, either at the beginning of ART or after dropping out, may use several concomitant medications such as ART, prophylaxis, and symptomatic medications, which may interfere with their treatment.

The use of immunosuppressants is a relevant risk factor for the reactivation of infection. According to the WHO guidelines, all individuals who start treatment with TNF-α antagonists should be screened for LTBI and, if documented, undertake treatment [2]. TNF-α is known to play a critical role in the control of the disease, increasing the phagocytic capacity of the macrophages and intracellular death of MTB through synergistic activity with interferon gamma, in addition to maintaining the formation and function of the granuloma in cases of latent infection. MTB activation may occur in individuals who have previously been exposed to the bacillus and those with latent infection [19]. The incidence of active TB in individuals who were treated using anti-TNF-α therapy was previously reported in a meta-analysis involving 11,879 people, identifying an incidence rate 1.94 (1.10–3.44) times higher in these individuals [19].

In the LTBI assessment, the most frequently performed diagnostic test was the TT, as it was the common choice for screening in Brazil’s public health system due to its greater accessibility, lower cost, and lower need for instrumentation. In 2021, the Brazilian public health system began to recommend IGRA for PLHIV with a CD4^+^ TL count of >350 cells/mm^3^, children aged between 2 and 10 years who had been in contact with TB, and candidates for hematopoietic stem cell transplantation [20]. However, changes related to the incorporation of these diagnostic tests require time to be implemented, as they require the updating and training of the relevant professionals in relation to the new methodology, in addition to the insurance of the availability of the supplies and technical capacity, which hinders the wide-scale realization of the IGRA.

A meta-analysis comparing the results of IGRA and TT in detecting and managing LTBI in a total of 50,592 individuals who had undergone these tests showed that positive results based on IGRA had a greater capacity to detect those at a higher risk of progression to active TB compared with the TT, indicating the greater clinical benefits of preventive treatment for LTBI in these patients [21].

Regarding imaging, patients with LTBI generally have a normal radiological pattern, as we found in this study, where 77.5% of the patients had normal plain chest radiography or computed tomography results. Ruling out active TB is usually necessary for patients with classic TB imaging alterations and respiratory or systemic symptoms. In this context, individualized evaluation and accurate diagnostic tests are essential, including acid-fast smears, rapid molecular tests, and mycobacterial cultures of the sputum or other biological specimens, before initiating LTBT. Here, these were performed on 24 patients [22].

The preferred treatment regimens recommended in Brazil up to 2021 were 9INH, 6INH, and 4RMP [6,23]. In terms of effectiveness, the adopted schemes are equivalent if they are completed. In the case of treatments with INH, there is a lower chance of developing TB when using the 9INH regimen, but the longer duration of the treatment and occurrence of adverse effects, especially hepatotoxicity, are treatment limitations. In contrast, regimens using rifampicin are associated with a lower risk of hepatotoxicity and superior cost–benefit ratio compared with those using INH [11,15,23]. However, the wide range of rifampicin-related drug interactions, such as a decrease in the serum level of dolutegravir due to the induction of hepatic enzymatic complexes, limits its use in PLHIV.

A retrospective study of LTBT conducted in the United States, which evaluated the 9INH, 4RMP, and 3HP regimens, showed similar treatment completion rates between the 4RMP and 3HP regimen groups (85.4% and 85.1%, respectively), with both regimens having higher completeness rates than the 9INH regimen (51.8%) [24]. Another similar study conducted in nine countries, including Brazil, with >6800 participants, showed that the 4RMP regimen was not inferior to the 9INH scheme in preventing active TB [25]. These data indicate that rifamycin family drug-based regimens have a shorter duration and lower incidence of collateral effects, including lower hepatotoxicity rates and similar efficacy to regimens with INH alone, and are associated with higher treatment adherence [4].

In this study, the mono-therapeutic regimens with INH (6INH and 9INH) showed completion rates no higher than 40%. In contrast, four patients who used the 4RMP regimen completed the proposed treatment. Despite the low number, these data suggest that regimens with a shorter duration and better tolerability, such as the proposed 3HP treatment in Brazil, may increase the rate of LTBT completeness.

Improving the availability of diagnostic tests can increase the rate of LTBI detection. However, the continuation of education and regular interventions by the healthcare system are necessary to avoid losses in the cascade of care [26].

State-level data concerning the TB control program suggest higher completion rates than those found in our study, with an average of 56% and a dropout rate of 13.4% from 2017 to 2019 [27]. A possible explanation for this higher rate of completeness could be the profile of the patients notified and treated through primary and secondary care, which included children and other TB contacts. However, our study evaluated people from specialized centers with concomitant comorbidities and specific treatments that could compromise the adherence to polypharmacy. In addition, the supervised treatment rates in these cases were not disclosed.

Up to 2021, a complicating factor in the local setting, particularly in the case of PLHIV, was the monthly dispensation of LTBT, whereas antiretrovirals had quarterly dispensations, resulting in the need for multiple visits to the pharmacy service. Dispensing both treatments for 3 months could act as a better way of facilitating adherence. However, as the 3HP regime was only introduced in Brazil in October 2021, an evaluation remains to be reported.

Adverse events related to the treatment were less frequent than the rates reported in other studies. One study compared different LTBTs and found that gastrointestinal intolerance, also observed in our study, was the second most common adverse event after exanthema [28]. In the same study, the most common adverse reaction in patients treated with 9INH was hepatotoxicity, which was reported in only one patient in our study. Another study including adults who received treatment with INH or RMP reported hepatotoxicity followed by gastrointestinal symptoms as the most common events [29]. Therefore, the lower occurrence of adverse effects observed in our study may reflect a bias in the data collection, especially in relation to the secondary data. However, the adverse effects were not responsible for treatment discontinuation or death.

Our study had some limitations. Its retrospective design may have compromised the quality of some of the collected data, as it may not have been sufficiently informative or accurate, in addition to the fact that there was missing data in the medical records. However, university hospitals are seeking to minimize this issue by checking all the data entered into their medical records. Furthermore, in 2017, 2018, and 2019, the notification forms lacked standardization, with several modifications, thus introducing some uncertainty into the final numbers for some variables, such as ethnicity, the education level, and active TB exclusion.

A further limitation was that this was a single-center study with no randomized sample, which may have resulted in selection bias. However, our institution is a tertiary hospital treating patients in Central-West Brazil, as well as patients from areas of North and Northeast Brazil. Finally, the reduced sample size may have contributed to the absence of statistically significant associations between the variables and outcomes. Therefore, these findings should be interpreted with caution and cannot be fully extrapolated to the state or national population.

However, the use of detailed clinical and laboratory data and pharmaceutical dispensing records, in relation to information on the outcomes, such as death and the development of active TB, was a strength of our study. This study also provided new data on local epidemiology, especially for the targeted populations. In the case of centers with similar profiles of the patients, our data are likely to be consistent in relation to LTBT, specifically in the immunocompromised population. Therefore, this study may help develop appropriate and effective strategies to reduce incompleteness rates.

## 5. Conclusions

In this study of LTBT in a specialized tertiary care service in Central-West Brazil (2017–2019), it was found that men aged between 30 and 60 years, those living with HIV, and those with CD4^+^ TL of < 350 cells/mm^3^ accounted for most of the population treated for LTBI. The TT was the first choice for screening, possibly because of its greater accessibility and lower costs. Even without diagnostic tests, certain groups with impaired immune systems, such as PLHIV and patients using immunosuppressants, can benefit from active TB prevention.

The 6INH and 9INH regimens had high dropout rates, reaching 65%. Therefore, shorter duration regimens such as 3HP and 4RMP may be promising alternatives in Brazil because of their similar efficacy and greater tolerability. PLHIV with severe immunosuppression would likely to benefit from the concomitant dispensation of ART and LTBT, in addition to a greater degree of directly observed treatment to minimize dropout, manage the patients’ polypharmacy challenges, and reinforce adherence.

This study provides evidence-based findings that can help to guide public health policies and support groups and to identify more appropriate follow-up strategies for patients at a higher risk of therapy dropout, thereby minimizing the incidence of active TB. Further studies are required to evaluate the extent of the improvements in LTBI screening and the possible increases in treatment completeness rates in Brazil following the recent availability of the IGRA test and 3HP regime in the public health system.

## Figures and Tables

**Table 1:** Clinical and epidemiological characteristics, examinations, therapeutic regimens, and outcomes of patients notified of an LTBT diagnosis in a Brazilian tertiary center from 2017 to 2019.

Variables	Total LTBT (*n* = 79) n (%)	* Complete Treatment (*n* = 30, 38%) n (%)	Incomplete Treatment (*n* = 49, 62%) n (%)	*p*
**Male sex (*n* = 54)**	54 (68.4)	23 (42.6)	31 (57.4)	0.160
**Age (years) (*n* = 79)—median (IQR)**	40 (30–51)	40 (28–52)	40 (30–51)	0.973
<30	21 (26.6)	9 (42.9)	12 (57.1)	
30–60	56 (70.9)	20 (35.7)	36 (64.3)	
>60	2 (2.5)	1 (50.0)	1 (50)	
**Self-reported race/ethnicity (*n* = 55)**				0.790
White	26 (47.3)	9 (34.6)	17 (65.4)	
Mixed race	21 (38.2)	9 (42.9)	12 (57.1)	
Black	8 (14.5)	4 (50.0)	4 (50.0)	
**Years of education (*n* = 43)**				0.159
≤12 years	34 (79.1)	16 (47.1)	18 (52.9)	
>12 years	9 (20.9)	3 (33.3)	6 (66.7)	
**Type of entrance (*n* = 78)**				0.104
New case	73 (92.4)	26 (35.6)	47 (64.4)	
Re-entry following a change in the treatment scheme	1 (1.3)	0 (0.0)	1 (100.0)	
**Risk factors for active TB (*n* = 79)**				
HIV infection	65 (82.3)	24 (36.9)	41 (63.1)	0.449
Immunosuppressants	12 (15.2)	6 (50.0)	6 (50.0)	0.268
Active smoking	12 (15.2)	4 (33.3)	8 (66.7)	0.719
DM	4 (5.1)	3 (75.0)	1 (25.0)	0.151
Chronic kidney disease	3 (3.8)	2 (66.7)	1 (33.3)	0.321
**CD4^+^ TL—median (IQR) (*n* = 65)**	257 (105–313)	251 (149–333)	239 (68–312)	0.623
≤350 cells/mm^3^	55 (84.6)	35 (63.6)	20 (36.4)	1.000
>350 cells/mm^3^	10 (15.4)	6 (60)	4 (40)	
**ART (*n* = 62)**				0.121
TDF + 3TC + DTG	44 (71.0)	19 (43.2)	25 (56.8)	
Other	18 (29.0)	4 (22.2)	14 (77.8)	
**BCG vaccine (*n* = 79)**				0.772
Yes	49 (62.0)	18 (36.7)	31 (63.3)	
No/not known	30 (38.0)	12 (40.0)	18 (60.0)	
**Plain chest radiograph description** **(*n* = 57)**				0.777
Normal	45 (78.9)	17 (37.8)	28 (62.2)	
Altered	12 (21.1)	4 (33.3)	8 (66.7)	
**Ruling out active TB (*n* = 79)**				0.534
Yes	22 (27.8)	8 (36.4)	14 (63.6)	
Ignored/unrealized	57 (72.2)	22 (38.6)	35 (61.4)	
**TT result (*n* = 42)—median (IQR)**	7 (0–13)	9.5 (0–14)	6 (0–10)	0.641
**Recommendation for LTBT (*n* = 79)**				0.500
No TT or IGRA	59 (74.7)	22 (37.3)	37 (62.7)	
TT > 5 mm or IGRA-positive	18 (22.8)	7 (38.9)	11 (61.1)	
TT >10 mm or IGRA-positive	2 (2.5)	1 (50.0)	1 (50.0)	
**Proposed treatment (*n* = 79)**				0.161
INH 300 mg, 9 months	61 (77.2)	21 (34.4)	40 (65.6)	0.009
INH 300 mg, 6 months	14 (17.7)	5 (35.7)	9 (64.3)	
RMP 600 mg, 4 months	4 (5.1)	4 (100.0)	0 (0.0)	
**Drug dispensing median (IQR)**	145 (90–195)	210 (180–270)	99 (90–120)	0.000
**Adverse effects (*n* = 15)**	15 (19)	5 (33.3)	10 (66.7)	0.681

* Complete “intention-to-treat” treatment reached the minimum goal of the INH regimen (180 doses) or RMP (120 doses); ART, antiretroviral therapy; BCG, Bacillus Calmette–Guérin; CD4^+^ TL, CD4^+^ T lymphocytes; DM, diabetes mellitus; DTG, dolutegravir; IGRA, interferon gamma release assay; INH, isoniazid; IQR, interquartile range; RMP, rifampicin; TB, tuberculosis; TDF, tenofovir; TT, tuberculin test; 3TC, lamivudine.

## Data Availability

The data presented in this study are available upon request from the corresponding author at http://doi.org/10.6084/m9.figshare.21347565.
